# Phytochemical profiling, *in vitro* biological activity, docking studies, and cytotoxicity assessments of *Rondeletia odorata* Jacquin: An unexplored plant of the coffee family

**DOI:** 10.3389/fchem.2022.1017577

**Published:** 2022-11-11

**Authors:** Anjum Khursheed, Saeed Ahmad, Muhammad Saleem, Kashif-ur-Rehman Khan, Jallat Khan, Ilkay Erdogan Orhan, Nurten Abaci, Muhammad Imran, Saba Tauseef, Reaz Uddin, Mirza Arfan Yawer, Muhammad Imran Tousif, Suvash Chandra Ojha, Umair Khurshid

**Affiliations:** ^1^ Department of Pharmaceutical Chemistry, Faculty of Pharmacy, The Islamia University of Bahawalpur, Bahawalpur, Pakistan; ^2^ Institute of Chemistry, Baghdad-ul-Jadeed Campus, The Islamia University of Bahawalpur, Bahawalpur, Pakistan; ^3^ Faculty of Natural, Health, Humanities and Social and Applied Sciences, Institute of Chemistry, Khwaja Fareed University of Engineering and Information Technology, Rahim Yar Khan, Pakistan; ^4^ Department of Pharmacognosy, Faculty of Pharmacy, Gazi University, Ankara, Türkiye; ^5^ Department of Chemistry, College of Science, King Khalid University, Abha, Saudi Arabia; ^6^ Dr. Panjwani Center for Molecular Medicine and Drug Research, International Center for Chemical and Biological Sciences, University of Karachi, Karachi, Pakistan; ^7^ Department of Chemistry, Division of Science and Technology, University of Education Lahore, Lahore, Pakistan; ^8^ Department of Infectious Diseases, The Affiliated Hospital of Southwest Medical University, Luzhou, China; ^9^ Southwest Medical University, Luzhou, China

**Keywords:** *Rondeletia odorata*, phytochemical profiling, *in vitro* biological activity, docking studies, cytotoxicity assessments

## Abstract

*Rondeletia odorata* Jacquin is a flowering plant that belongs to the coffee family. As a rich source of polyphenols with significant antioxidant potential, *R. odorata* may have health benefits. Therefore, in the current work, ethanolic extract of aerial parts and its n-hexane, ethyl acetate, and n-butanol soluble fractions were analyzed for their antioxidant potential and various enzyme inhibition properties. The total phenolic and flavonoid contents of the crude ethanol extract (ROE) and its n-hexane (ROH), ethyl acetate (ROEA), and n-butanol (ROB) fractions were determined spectrophotometrically, while metabolic profiling was established through UHPLC-MS analysis, which revealed the presence of 58 phytochemicals. Total phenolic and flavonoid contents of ROE extract were measured as 51.92 mg GA.Eq./g of dry extract and 52.35 mg Qu.Eq./g of the dry extract, respectively. In the DPPH radical scavenging activity assay, ROE and ROEA showed the highest potential with values of 62.13 ± 0.62 and 76.31% ± 1.86%, respectively, comparable to quercetin (80.89% ± 0.54%). Similarly, in the FRAP assay, the same pattern of the activity was observed with ROE and ROEA, which displayed absorbance values of 1.32 ± 0.01 and 0.80 ± 0.02 at 700 nm, respectively, which are comparable (1.76 ± 0.02) with the reference compound quercetin, whereas the ROH showed maximum metal-chelating capacity (62.61% ± 1.01%) among all extracts and fractions. Antibacterial activity assay indicated that the ROEA fraction was the most active against *Serratia marcescens*, *Stenotrophomonas maltophilia*, *Bacillus subtilis*, *Klebsiella pneumonia*, and *Staphylococcus aureus*, while the rest of the fractions showed good to moderate activity. Enzyme inhibition assays showed that ROEA fraction exhibited the highest activity with IC_50_ values of 2.78 ± 0.42 and 3.95 ± 0.13 mg/mL against urease and carbonic anhydrase (CA), respectively. Furthermore, the docking studies of some of the major compounds identified in the extract revealed a strong correlation with their inhibitory activity. All extracts and fractions were also tested for their thrombolytic activity, and the ROB fraction showed a notable potential. Antiviral assay led to remarkable outcomes. Thus, it can be inferred that aerial parts of *R. odorata* are potential sources of bioactive components with several significant pharmacological activities.

## 1 Introduction

Natural products have played a crucial role in medicine and health throughout the course of human evolution. Natural remedies have frequently been used for treating illnesses and wounds since our earliest ancestors chewed particular herbs to relieve pain or wrapped leaves around wounds to improve healing. In the modern era, researchers can now properly explain the biological impact of natural substances on individuals and find potential synergies thanks to modern chemistry and biology. This has enormous promise for the development of cutting-edge therapies for a variety of crippling illnesses. Therefore, it is crucial to carry out continuous natural source screening for the development of therapies for a number of debilitating diseases (Ji et al., 2009).

Among countless medicinal herbs, *Rondeletia odorata* Jacquin (Syn: *R. speciosa* Lodd; *R. brilliantissima* Hend; *R. coccinea* and *R. obovata* L.) (List, 2013) of the family Rubiaceae is an evergreen shrub, native to Cuba and Panama. In Pakistan, it is grown as an ornamental plant. Common names for the plant are “sweet-smelling *Rondeletia*” or “fragrant Panama rose” (Mazza, 2009). A literature search revealed that Rubiaceous plants produce new potential metabolites and therapeutic prototypes (Martins and Nunez, 2015). Phenolics, anthraquinones, alkaloids, coumarins, flavonoids, and terpenes are only a few of the secondary metabolites they produce, many of which have pharmacological effects (Heitzman et al., 2005). Furthermore, the Rubiaceae plants show high antioxidant activity (Mavi et al., 2004; Soobrattee et al., 2008; Lakić et al., 2010; Torey et al., 2010), which is due to its secondary metabolites. *Rondeletia* is an important genus of Rubiaceae, which is used traditionally in different countries around the world (Michel et al., 2007; Carlomagno et al., 2015). *R. panamensis* DC. is a Panamanian plant, which produces cytotoxic diterpenes (Koike et al., 1980). According to the literature, *Rondeletia* displays interspecific variation, with some species having negative results for alkaloids and others yielding positive results for alkaloids of varying concentrations, and this variation is attributed to different possible metabolic pathways to produce different compounds. This complication makes biological screening more intriguing for *Rondeletia* plants (Soto-Sobenis et al., 2001). Despite its biological importance and interesting metabolic behavior, the *Rondeletia* genus is underexplored. Likewise, no report so far has been found in the literature on the phytochemical and biological screening of *R. odorata*. Therefore, based on various folkloric uses of the plants from the coffee family and their reported biological activities, we designed the current study on *R. odorata* to identify its phytochemicals and evaluate it for medicinal potential as a component of nutraceuticals and functional foods.

## 2 Materials and methods

### 2.1 Collection of the plant material and identification

The aerial parts of the plant were collected in February 2019, near Pattoki Bypass, Kasur, Punjab, Pakistan, where it was grown as an ornamental plant. It was authenticated by Dr. Ghulam Sarwar from the Department of Botany, The Islamia University of Bahawalpur, and was deposited in the herbarium with specimen No. 167/Botany.

### 2.2 Extraction and fractionation

Plant material was first rinsed with distilled water to remove dirt and shade-dried for 15 days and got 2.5 kg dried plant material. The dried material was extracted with aqueous ethanol (80%) by maceration for a period of 15 days with occasional vigorous shaking. The filtrate was concentrated using a rotary evaporator. The crude extract (85 g) obtained was suspended in 250 ml of distilled water and successively fractionated with solvents of increasing polarity such as n-hexane, ethyl acetate, and n-butanol to respective fractions denoted as ROH (15 g), ROEA (20 g), and ROB (30 g). All fractions obtained were stored at 4°C for further studies.

### 2.3 Measurement of total phenolic and flavonoid contents

The total phenolic contents (TPCs) of the fractions were measured using the Folin–Ciocalteu reagent, while their total flavonoid contents (TFCs) were assessed using the AlCl_3_ colorimetric method (Slinkard and Singleton, 1977; Zengin et al., 2016). In these analyses, the phenolic and flavonoid contents were presented as equivalents of gallic acid (mg GA.Eq./g dried extract) and quercetin (mg Qu.Eq./g of dry extract), respectively.

### 2.4 UHPLC-MS analysis of the ethanolic extract (ROE)

Phytochemical profiling was accomplished through UHPLC-MS analysis, which was performed on an Agilent 1290 infinity UHPLC system coupled with an Agilent 6520 accurate mass Q-TOF mass spectrometer with a dual ESI source. For metabolite separation, an Agilent Zorbax Eclipse XDB-C18 column (2.1150 mm, 3.5 m) was employed. Mobile phase A was a 0.1% formic acid solution in water, and mobile phase B was a 0.1% formic acid solution in acetonitrile. A Rheodyne-type injector was used to load 1.0 μL of injection volume, and the experiment was run with a flow rate of 0.5 ml/min and an acquisition time of 25 min. The electrospray ion source was used to perform 100–1000 MS scans in the positive mode. With a flow rate of 25 and 600 L/h, respectively, and a drying gas temperature of 350°C, nitrogen gas was employed for nebulizing and drying. The capillary voltage for analysis was 3500 V, whereas the fragmentation voltage was tuned at 125 V. The secondary metabolites were identified using the METLIN database (Khan et al., 2019).

### 2.5 Antibacterial assays (broth microliter plate dilution method)

Two Gram-positive strains, *Staphylococcus aureus* and *Bacillus subtilis*, and eight Gram-negative strains, *Escherichia coli*, *Citrobacter koseri*, *Klebsiella pneumonia*, *Pseudomonas aeruginosa, Proteus mirabilis, Morganella morganii, Stenotrophomonas maltophilia,* and *Serratia marcescens*, were provided by the Department of Medical Laboratory Sciences, Bahawal Victoria Hospital, Bahawalpur, Pakistan. These bacterial strains were used to evaluate the antibacterial potential of the extracts through the broth microtiter plate dilution method in sterilized 96-well ELISA microplates (Rehman and Ahmad, 2019). The total assay mixture volume was kept at 150 μL in each well, which constituted 75 μL of extract/fraction solutions (concentration of 5 mg/ml dried extract/fractions in DMSO each) and 75 μL of bacterium inoculums. The absorbance of all of these clear solutions was measured at 540 nm on a BioTek Synergy HT ELISA microplate reader and was considered as pre-read. Then, 96-well ELISA microplates were incubated for 24 h at 37°C, and again the absorbance was determined at 540 nm, which was regarded as after-read. The difference of after-read subtracted by pre-read was attributed to the bacterial growth inhibition index. Ceftriaxone (1 mg/ml in DMSO) was employed as the + ve control, whereas distilled water was used as the -ve control. Inhibition (%) of bacterial strains was enumerated by the formula given below:

Inhibition (%) of bacterial strain=[(S.V. of test solution / S.V. of blank)]∗ 100.

Serial dilutions (2,500, 1250, 625, 312.5, 156.2, and 78.12 μg/ml) of the extract/fractions and ceftriaxone solution (reference drug) were prepared to estimate their antibacterial potential by measuring MIC_50_, which was obtained using software “EZ-Fit™ Perrella Scientific Amherst United States”. All of the experiments were carried out in triplicate.

### 2.6 Antioxidant activity assays

#### 2.6.1 DPPH radical scavenging activity assay

The antioxidant capacity of the corresponding samples was computed from the bleaching property of the violet-colored methanol solution of 2,2-diphenyl-1-picrylhydrazyl (DPPH, Sigma, United States). The stable DPPH radical scavenging activity was determined by the method of Hatano et al. (1988) with minor modifications (Barros et al., 2007). Next, 10 µL of the samples/reference dissolved in EtOH was transferred to 96-well plates. Then, 90 µL of DPPH solution (1.5 × 10–4 M) prepared in EtOH was added to each well using a multichannel pipette (Eppendorf Research, Germany). The remaining quantity of DPPH was then measured by spectrophotometry using an ELISA microplate reader (Molecular Devices, SpectraMax i3x microplate reader, United States) at 515 nm following incubation at 37°C for 30 min. The results were compared to quercetin (1000 μg/ml, Sigma, United States), which was used as the reference. Measurements were taken in triplicate.

#### 2.6.2 Ferric-reducing antioxidant power assay

The ferric-reducing ability of the samples was examined using the Oyaizu assay with minor modifications to measure the antioxidant capability (Oyaizu, 1986). The assay was based on the reducing power of the conversion of ferric ion (Fe3+) to ferrous ion (Fe2+), which creates a blue complex (Fe2+/TPTZ) that enhances absorbance at 700 nm. Briefly, 10 μL of the samples and reference in EtOH (96%) were transferred to a 96-well microplate and preincubated at 50°C for 20 min after being treated with 25 ml of phosphate buffer (pH 6.6) and 25 µL of [K3Fe(CN)6] (1%, w/v, Sigma, United States ). Then, 25 µL of trichloroacetic acid (10%, Sigma, United States), 85 µL of distilled water, and 17 µL of FeCl3 (0.1%, w/v) were added and incubated at room temperature for 30 min. The absorbance of the generated complex was measured at 700 nm using an ELISA microplate reader (Molecular Devices, SpectraMax i3x microplate reader, United States). Quercetin (1000 μg/ml, Sigma, United States) was the reference in this assay. The analysis was performed in triplicate.

#### 2.6.3 Metal-chelating activity assay

Carter’s modified approach was used to determine the metal-chelating impact of the samples, where the reference was ethylenediaminetetraacetic acid (EDTA, Sigma, United States) (Carter, 1971; Lantto et al., 2009). In brief, 20 μL of each sample and reference was incubated for 10 min at ambient temperature with EtOH (96%), 2 mM FeCl_2_ (Sigma, United States), and ferrozine (5 mM, Sigma, United States) solutions. The absorbance of the ferrozine-Fe2+ complex formed was measured at 562 nm using an ELISA microplate reader (Molecular Devices, SpectraMax i3x microplate reader, United States).

#### 2.6.4 Data processing for antioxidant activity assays

DPPH radical scavenging and metal-chelating activity assay findings of the samples were calculated as given below, then represented as percent inhibition (I%).

$$\%I=\frac{A_{blank}-A_{sample}}{A_{blank}}\times 100,$$

where A_blank_ denotes the absorbance of the control reaction (all reagents except the test sample) and A_sample_ denotes the absorbance of the samples/reference. Analyses were performed in triplicate, and the data were reported as averages with standard deviations (S.D.). FRAP assay was likewise performed in triplicate, and the higher absorbance of the reaction signified higher reducing power in this assay.

### 2.7 Enzyme inhibition assays

#### 2.7.1 Cholinesterase inhibition assays

Inhibitory activity of the extract/fractions against AChE and BChE was determined using a slightly modified version of Ellman’s method (1961). Electric eel AChE (type-VI-S, EC 3.1.1.7, Sigma) and equine serum BChE (EC 3.1.1.8, Sigma) were employed as the enzyme sources, while acetylthiocholine iodide and butyrylthiocholine chloride (Sigma, St. Louis, MO, United States) were used as reaction substrates. 5,5´-Dithio-bis(2-nitrobenzoic) acid (DTNB, Sigma, St. Louis, MO, United States) was used for measurement of the cholinesterase activity. First, 140 µL of 0.1 mM sodium phosphate buffer (pH 8.0) was added to the 96-well microplate with a multichannel automatic pipette (Eppendorf Research, Germany), and then 20 µL of the sample/EtOH (negative control) was added at dilutions ranging from 25 to 200 μg/ml. Then, 20 µL of 0.2 M AChE/BChE solution was added using a multichannel automatic pipette (Gilson Pipetman, France). After that, it was incubated at room temperature for 10 min. The reaction was started by adding 10 μL of 0.2 M acetylthiocholine iodide/butyrylthiocholine chloride as substrates to the 96-well microplate. Thiol esters used as substrates are hydrolyzed by AChE or BChE to release thiocholine. As a result of the reaction of thiocholine with DTNB, 2-nitro-5-thiobenzoate (TNB) is formed as the yellow product. The formation rate and color intensity of the product, which formed as a result of the reaction, were measured using an ELISA microplate reader (Molecular Devices, SpectraMax i3x microplate reader, United States) at a wavelength of 412 nm. Galanthamine hydrobromide (Sigma, United States) was used as the reference drug in both experiments. All experiments were performed in triplicate. Based on a comparison of rates of enzyme reaction between the sample and the blank sample (ethanol in phosphate buffer, pH 8) using formula (1-S/E)*100, where E is enzyme activity without test sample and S is enzyme activity with the test sample, we determined the percentage of inhibition of AChE and BChE. GraphPad Prism 6.01 was used to compute IC_50_ values of understudy aerial extract/fractions.

#### 2.7.2 Urease inhibition assay

Urease inhibition assay was carried out as detailed by Bashir et al. (2017) with minor alterations. The total volume of the assay mixture was 200 μL, which contained 15 μL of urease enzyme solution (0.25 mg of urease in 1 ml of 1M phosphate buffer; pH 7), 15 μL of 1M phosphate buffer solution (pH 7), and 15 μL of extract/fraction solutions (5 mg/ml each). All solutions were poured into sterilized 96-well ELISA microplates and incubated for 15 min at 37°C. Urea solution (40 μL) was then added as the reaction substrate, and the ELISA plate was reincubated under similar conditions. After incubation, the pre-read was measured by taking absorbance at a wavelength of 630 nm. After taking pre-read, a 45 μL volume of phenol solution with a 70 μL volume of alkali reagents was mixed in the reaction mixture. The microplate reaction mixture was incubated again for 50 min at 37°C. Absorbance was taken again at a wavelength of 630 nm and was regarded as post-read. Thiourea was taken as a reference, while methanol was considered as a control. Percentage inhibitions (%) by various test solutions were measured by the formula given below:

Inhibition (%) of urease = 100—[(S.V. of control solution—S.V. of test solution)/S.V. of control solution] ∗ 100.

IC_50_ values of the extract/fractions were determined by making various dilutions of different concentrations (2,500, 1250, 625, 312.5, 156.2, and 78.12 μg/ml) of the first concentration (5,000 μg/ml). All of the experiments were performed in triplicate.

#### 2.7.3 Carbonic anhydrase inhibition assay

CA inhibition procedure was performed as stated in the method by (Ashiq et al., 2017) with minute modifications. Acetazolamide was taken as the reference. The total assay volume was 200 μL. Tris-HEPES buffer of pH 7.4 (140 μL) with 20 μL of CA (0.2 mg of CA in 1 ml of deionized water) and 20 μL of each sample solution (concentration of 5,000 μg/ml each) were mixed in sterilized 96-well ELISA microplates and incubated for 15 min at 25°C. Absorbance was noted at 400 nm as pre-read. Then, 20 μL of substrate, which was 4-nitrophenol acetate (0.7 mM), was added, the microplate was reincubated at the same temperature for 30 min, and the post-read was determined at the same wavelength. All of the experimentation was carried out in triplicates, and Percentage (%) inhibition of CA was determined by the formula given below:

Inhibition (%) of CA = [100 − (S.V. of control solution—S.V. of test solution)/(S.V. of control solution)] ∗ 100.

IC_50_ values of the extract/fractions were determined by making various dilutions of different concentrations (2,500, 1250, 625, 312.5, 156.2, and 78.12 μg/ml) of the first concentration (5,000 μg/ml). All of the experiments were performed in triplicate.

#### 2.7.4 Tyrosinase inhibition assay

Inhibition of tyrosinase (EC 1.14.1.8.1, 30 U, mushroom tyrosinase, Sigma) was determined using the modified dopachrome method with L-DOPA as substrate (Masuda et al., 2005). The assays were conducted in a 96-well microplate using an ELISA microplate reader (VersaMax Molecular Devices, United States) to measure absorbance at 475 nm. An aliquot of the extracts dissolved in DMSO with 80 μL of phosphate buffer (pH 6.8), 40 μL of tyrosinase, and 40 μL of L-DOPA were put in each well. Results were compared with the control (DMSO). Alpha-kojic acid (Sigma, St. Louis, MO, United States) was used as the reference.

### 2.8 Hemolytic assay

The hemolytic effect of the extract/fractions was evaluated using (Diaconu et al., 2020) with slight modifications. 10 ml of blood from human volunteers was collected and then poured into a top-screwed EDTA tube and centrifuged for 5 min. The upper layer was separated out, and red blood cells were washed many times with 10 ml of cooled sterilized isotonic phosphate buffer saline (PBS) having pH 7.4. Washed cells were again suspended in 20 ml of PBS, and the test samples (1 mg/ml dried extract/fractions in methanol) each were added to this mixture separately and incubated at 37°C for 60 min. The hemolysis rate was calculated by determining the absorbance of hemoglobin present in the supernatant at the wavelength of 540 nm. Triton X-100 (0.1%) was used as the positive control and PBS as the negative control. Hemolysis (%) was calculated through their absorbance (A) using the following formula:

$$Hemolysis\;\left(\%\right)=\left(A_{sample}\;–\;A_{negative\;control}\right)/A_{positive\;control}\times 100.$$

### 2.9 Thrombolytic assay

To perform thrombolytic assay with the extracts, healthy human volunteers (who did not have any history of undergoing anticoagulant and oral contraceptive therapy from the last 7 days) were selected. Venous blood (5 ml) was collected from each volunteer and poured into preweighed and sterile specific centrifuge tubes. Incubation of these tubes was carried out at 37°C for 45 min. After the blood clot was formed, the entire fluid from each centrifuge tube was discharged. Blood clot weight was determined by subtracting the weight of the empty centrifuge tube from the one containing the clot. Streptokinase was used as the reference, which was prepared by diluting the commercially available streptokinase (1,500,000 I.U.) injection with 5 ml of sterilized water. Then, 100 µL of streptokinase (30,000 I.U) was used as the positive control, while 100 µL of distilled water was the negative control. Each extract/fraction was added to the centrifuge tube containing the clot, and then all tubes were incubated at 37°C for 90 min. After that, examination of clot lysis was performed and all of the extra fluid from the tubes was discarded. The centrifuge tubes were again weighed to observe the weight variation subsequent to clot lysis (Saleem et al., 2015). The percentage of clot lysis was determined using the following formula:

Clot lysis (%)=(Reduced clot weight/Weight of clot)×100.

### 2.10 Antiviral assay

#### 2.10.1 Inoculation of viruses in chicken embryonated eggs (cultivation of viral strains)

Chicken embryonated eggs are the most widely used medium for inoculation studies as the inoculive-stock is very valuable. The eggs may be used for the initial growth of viruses, propagation, and development of new vaccines. The excellent yield of viruses from chicken eggs has made them the most used medium for viral culturing. Poultry eggs are easily available, easy to handle, need no extra care, are least expensive, can be used in aseptic conditions, and require little space . This makes them the best source of studies on viral inoculations. During the incubation period, the virus replicates and gets accumulated in the chorio-allantoic membrane fluid. In 7–11-day-old embryonated eggs of chicken, all viral strains were cultured. From the Government Poultry Farm, Model Town A Bahawalpur, pathogen-free eggs were taken. With the help of a 5 cc-syringe, the viral strains were inoculated through the chorio-allantoic route. The eggs were sterilized with 70% EtOH, and a hole was made with the help of a sterilized common pin. After inoculation, the hole was closed with melted wax. The eggs were incubated at 37°C for 48–72 h. The allantoic fluid was collected and exposed to hemagglutination (HA) and indirect hemagglutination (IHA) to assess the titers of virus. Different sites can be used for viral inoculation, that is, chorio-allantoic membrane, allantoic cavity, amniotic cavity, and yolk sac (Andleeb et al., 2020).

#### 2.10.2 Hemagglutination (HA) test

Alsever solution (20 ml) is poured into a test tube, and after that, 5 ml of fresh chicken blood is added to it. Blood (10 ml) was centrifuged at 4,000 rpm for 5 min, and the supernatant was discarded. The process was repeated three times for further purification and to obtain a better result. RBC solution (1%) was prepared by adding 10 µL of packed RBCs into 1 ml of PBS solution (pH 7.4) placed in Eppendorf tubes. The tubes were shaken gently to avoid any kind of precipitation. PBS (50 µL) was added in each well of a 96-well round-bottom microtiter plate. Then, 50 µL of viral sample or allantoic fluid was added in the first column and serially diluted to the 11th well. The 12th well was left as a negative control (PBS only). Then, 50 µL of 1% RBC solution was added to each well and the plate was incubated for 2–3 h at 37°C. Red dots at the bottoms of the wells indicated positive results, while a uniform reddish color pointed out negative results. The highest dilution number was the HA titer that showed a positive result. The test was used for testing the titer of NDV, IBV, and H9N2 (Harazem et al., 2019).

### 2.11 Docking experiments

Six compounds selected for inhibitory activity against urease and carbonic anhydrase were drawn in ChemDraw 3D (Mills, 2006) and optimized, while energy was minimized using MMFF94. The stable energy-minimized conformations for the compounds were used for docking studies. The 3D structures for urease (PDB I.D. 4H9M) and carbonic anhydrase (PDB I.D. 3DC3) were retrieved from the RCSB Protein Data Bank (PDB) (Sussman et al., 1998). Prior to docking studies, co-crystallized ligands, water, and small molecules were removed from the proteins. Docking was performed using AutoDock 4.2 software. The protonation state of both proteins was satisfied by adding polar hydrogen. The Kollman charges were also added to both the proteins, while Gasteiger charges were added to ligands using AutoDock. The proteins and ligands were saved in PDBQT format as separate files. The position of the grid box was adjusted so that it was centered on the co-crystallized ligand, while dimensions were set as 40*40*40 A° in x, y, and z coordinates. The docking protocols were set for 250 runs using the Lamarckian genetic algorithm.

### 2.12 Statistical analysis

Whole experimentation was carried out in triplicate, and the results were represented as average ± S.D. (standard deviation). One-way ANOVA was applied pursued by the LSD test for comparing various study groups. Statistix version 8.1 was used for analyzing the results.

## 3 Results and discussion

### 3.1 Percentage yields of extracts

The percentage of the extract recovery was estimated for different solvent extraction obtained through solid–liquid extraction as given in (Table 1). Maximum extract yields among different solvents, for example, 80% ethanol–water, n-hexane, ethyl acetate, and n-butanol, were observed for 80% ethanol–water extract with 8.6% ± 0.2%, followed by 5.4% ± 0.3% for n-butanol, 1.6% ± 0.5% for ethyl acetate, and 1.3% ± 0.1% for n-hexane. The current scientific assessment has validated that the recovery of bioactive constituents and extraction yield is totally reliant on extraction time, extraction technique, and solvent polarity (Zohra et al., 2019).

**TABLE 1:** Results of % age extractive yield for ground plant material using different solvents.

Plant samples	Percentage age of extractive value
ROE	8.6 ± 0.2^a^
ROH	1.3 ± 0.1^d^
ROEA	1.6 ± 0.5^c^
ROB	5.4 ± 0.3^b^

Experimentation was carried out in triplicate, and results are represented by [mean ± S.D.] with different superscripts a–d showing that all of the values were momentously different from one another (*p* ≤ 0.05). ROE, hydroethanolic fraction; ROH = n-hexane fraction; ROEA, ethyl acetate fraction; and ROB = n-butanol fraction.

### 3.2 Total bioactive contents and UHPLC-MS analysis

In the present study, crude aq. ethanolic extracts of *R. odorata* and its fractions were estimated for their total phenolic and flavonoid contents. It was observed that overall the extracts contained more flavonoid content than the phenolics. The ROEA fraction comprised of the highest amount of phenolics and flavonoids (246.48 mg GA.Eq./g and 300 mg Qu.Eq./g of dried extract), followed by ROB (36.32 mg GA.Eq./g and 136.47 mg Qu.Eq./g of dried extract), ROE (51.92 mg GA.Eq./g and 52.35 mg Qu.Eq./g of dried extract), and ROH fraction (6.48 mg GA.Eq./g and 161.17 mg QEq/g extract) (Table 2). These results indicated that ethyl acetate could extract the maximum amount of phenolics and flavonoids, which is exactly in line with various literature reports (Hossain et al., 2019; Khan et al., 2019).

**TABLE 2:** Results of total phenolic and flavonoid contents of the *R. odorata* extract/fractions.

Sample codes	Total phenolic contents (mg GA.Eq./g of dry extract)	Total flavonoid contents (mg Qu.Eq./g of dry extract)
ROE	51.92	52.35
ROH	6.48	161.17
ROEA	246.48	300
ROB	36.32	136.47

#### 3.2.1 Secondary metabolites profiling through UHPLC MS analysis

To have a deep look into the metabolic profile of *R. odorata*, the crude aq. ethanolic extract (ROE) was subjected to UHPLC-MS analysis (Table 3), which led to the identification of 58 compounds of alkaloid, phenolic, flavonoid, terpenoid, and steroid classes. The identified compounds include nigellimine N-oxide, an alkaloid, which is the main component isolated from the seeds of *Nigella sativa* (MALIK et al., 1985) and is known for its antioxidant, antimicrobial, anticancer, antidiabetic, and anti-inflammatory activities (Manoharan et al., 2021). Other identified alkaloids mostly belong to indole, quinoline, and isoquinoline subclasses, *viz.*, robustine, (R)-norreticuline, nepharadione A, nristolodione, piperolactam A, α,β-didehydrotryptophan, oxoaporphine, liriodenine, and atheroline. Piperolactam A was reported to have antiviral activity (Kothandan et al., 2021). These alkaloids are characteristic features of the coffee family, which make *R. odorata* important with respect to antioxidant, antibacterial, and antiviral activities. Furthermore, the presence of phenolics in a higher concentration along with flavonoids and withanolide makes *R. odorata* more important. In addition, docking studies of some of these metabolites against some enzymes substantiated their medicinal properties. According to these findings, *R. odorata* produces a variety of compounds and is not limited to a specific class of secondary metabolites. Therefore, it is concluded that *R. odorata* is a valuable herb with a wide range of bioactivities due to its chemodiversity.

**TABLE 3:** UHPLC-MS-based identification of secondary metabolites in ROE.

Sr No.	Analyte peak mass	Retention time	Area/height	Identified compounds	Class of compound	Molecular formula	Molecular mass
1	220.1000	1.35	6.83	Nigellimine N-oxide	Alkaloid	C12H13NO3	219.08
2	363.0467 (M + K+)	1.46	4.24	Mahaleboside	Coumarin	C15H16O8	324.28
3	245.0943 (M + NH4+)	1.49	5.93	Mukeic acid	Carbazole	C13H9NO3	227.21
4	217.0507	1.53	11.70	Norvisnagin	Phenolic	C12H8O4	216.19
5	371.1216 (M + Na+)	1.52	8.41	Trans-anhydrotephrostachin	Flavonoid	C22H20O4	348.40
6	393.1440	1.55	8.09	Shanzhiside	Terpene	C16H24O11	392.13
7	415.1276 (M + Na+)	1.53	9.52	Caryoptosidic acid	Terpene	C16H24O11	392.35
8	216.0689	1.64	10.63	Robustine	Alkaloid	C12H9NO3	215.20
9	242.0635	2.75	5.16	2,4-Dihydroxy-6,7-dimethoxy-2H-1,4-benzoxazin-3(4H)-one	Phyto	C10H11NO6	241.20
10	343.0808	2.75	7.78	8-Hydroxygalangin 7-methyl ether 8-acetate	Flavonoid	C18H14O7	342.073
11	163.0237 (M + CH3OH + H+)	3.03	8.26	2-Thiophenemethanethiol	Phyto	C5H6S2	130.2
12	273.0581	3.19	10.64	Fukiic acid	Phenolic	C11H12O8	272.21
13	197.0637	3.48	12.71	Gulonic acid	Phenolic	C6H12O7	196.16
14	185.0808	3.50	7.77	1-(3-Hydroxy-4-methoxyphenyl)-1,2-ethanediol	Phenolic	C9H12O4	184.19
15	230.0817	3.50	7.98	Fenamisal	Phenolic	C13H11NO3	229.23
16	300.0872	3.51	8.07	Avenanthramide 1c	Phenolic	C16H13NO5	299.28
17	244.0972	3.51	8.08	N-Desmethyltolmetin	Phyto		
18	227.0735 (M + CH3OH + H+)	3.79	6.97	D-Glucuronic acid	Sugar	C6H10O7	194.25
19	316.1553	3.83	7.36	(R)-Norreticuline	Alkaloid	C18H21NO4	315.4
20	297.0745 (M + Na+)	4.07	9.30	Guibourtinidol-4α-ol	Phenolic		
21	288.0664	4.33	7.85	Piperolactam A	Alkaloid	C16H11NO3	265.26
22	291.0657	4.33	7.36	6-Hydroxy-2'-methoxyflavone	Flavonoid	C16H12O4	268.26
23	225.0580	4.50	7.51	α, β-Didehydrotryptophan	Alkaloid	C11H10N	202.209
24	427.0956 (M + Na+)	4.51	7.47	Distemonatin	Flavonoid	C20H20O9	404.4
25	227.1096	4.54	8.77	Phenylmethyl benzeneacetate	Phenolic	C15H14O2	226.0994
26	197.0640	4.79	7.17	Gulonic acid	Phyto	C6H12O7	196.05
27	359.1472	4.62	6.79	2',4',5,7-Tetramethoxy-8-methylflavanone	Flavonoid	C20H22O6	358.14
28	387.1779	4.62	10.76	3,5-Di-O-methyl-8-prenylafzelechin-4β-ol	Flavonoid	C22H26O6	386.4
29	375.1208 (M + CH3OH + H+)	4.71	7.26	Glucocaffeic acid	Phenolic	C15H18O9	342.30
30	413.1173	4.79	6.73	(2E)-5,7-Dihydroxy-3,6-dimethoxy-4-oxo-2-phenyl-4H-1-benzopyran-8-yl ester 2-methyl-2-butenoic acid	Phenolic		
31	193.0693	4.79	6.70	Quinic acid	Phyto	C7H12O6	192.17
32	306.0762	4.82	7.19	Cepharadione A	Alkaloid	C18H11NO4	305.3
33	301.0861 (M + K+)	4.82	7.84	Dihydrosuberenol	Phenolics	C15H18O4	262.30
34	325.1177 (M + NH4+)	5.02	6.32	Aristolodione	Alkaloid	C18H13NO4	307.3
35	545.1708 (M + Na+)	5.02	6.86	Melampodinin	Terpenoid	C25H30O12	522.5
36	308.0919 (M + CH3OH + H+)	5.19	7.46	Liriodenine	Alkaloid	C17H9NO3	275.26
37	448.1919 (M + NH4+)	5.35	6.78	Aliarin 4'-methyl ether	Flavonoid	C23H26O8	430.4
38	313.0707	5.33	10.39	3',4,4'-Trihydroxypulvinone	Phenolics	C17H12O6	312.27
39	412.1937 (M + NH4+)	5.36	10.34	Gibberellin A28	Terpenoid	C20H26O8	394.4
40	313.0709	5.59	8.17	8-Hydroxy-3-methoxy-1-methylanthraquinone-2-carboxylic acid	Phenolic	C17H12O6	312.27
41	343.0805	5.54	9.55	8-Hydroxygalangin 7-methyl ether 8-acetate	Phenolic	C18H14O7	342.07
42	167.0549	5.62	9.02	Apionic acid	Phyto	C5H10O6	166.13
43	165.0744	5.51	9.12	α-L-fucose	Sugar	C6H12O5	164.16
44	347.1266 M + CH3OH + H+	6.07	9.60	2-Hydroxyphenylacetic acid O-β-D-glucoside	Phenolic	C14H18O8	314.29
45	338.0979	6.11	6.65	Atheroline	Alkaloid	C19H15NO5	337.3
46	163.0237	6.51	6.27	4-Hydroxy-2-oxo-glutaric acid	Sugar	C5H6O6	162.10
47	259.1349 M + CH3OH + H+	6.59	7.63	2-Phenylethyl benzoate	Phenolic	C15H14O2	226.27
48	425.3140	6.69	8.10	Norselic acid C	Steroid	C28H40O3	424.6
49	501.2110 M + H + Na2+	6.96	8.08	Senegin II	Saponin	C70H104O32	1457.6
50	407.2199	6.95	7.64	Erycristin	Isoflavonoid	C26H30O4	406.5
51	423.3009	7.32	6.77	Cholic acid methyl ester	Steroid	C25H42O5	422.6
52	667.3922	7.36	5.61	Lucyoside R	Saponin	C36H58O11	666.3979
53	666.3891 M + NH4+	7.35	8.11	Cytotrienin A	Phyto	C37H48N2O8	648.8
54	671.3452 M + Na+	7.35	6.04	Lyciumoside III	Terpenoids	C32H56O13	648.7792
55	470.3085	7.86	10.31	Methymycin	Macrolide	C25H43NO7	469.6
56	471.3103	8.29	8.46	Minabeolide-8	withanolide	C29H42O5	470.6
57	423.3009	8.28	7.43	Minabeolide-1	withanolide	C28H38O3	422.6
58	699.3232	8.95	5.90	Evasterioside A	Steroid	C33H55NaO12S	698.84

### 3.3 Antibacterial activity

The broth microtiter plate dilution method (BMPDM) (Balouiri et al., 2016) was used to study the antibacterial potential of the extract and fractions obtained from *R. odorata* aerial parts. BMPDM determines the minimal inhibitory concentration (MIC_50_) value, which is defined as the lowest concentration of antibacterial agent that inhibits the growth of tested microorganisms by 50% in microplates; the results are expressed in Table 4. In the present study, all extract/fractions were tested against two Gram-positive bacteria (*S. aureus* and *B. subtilis*) and eight Gram-negative bacterial strains (*K. pneumonia*, *M. morganii*, *S. maltophilia*, *E. coli*, *C. koseri*, *S. marcescens*, *P. aeruginosa*, and *P. mirabilis*). All extracts showed good results (Table 4) against bacterial strains, except *E. coli*, *C. koseri*, *P. aeruginosa*, and *P. mirabilis*. Results indicated that ROEA extract was active against maximum numbers of the bacterial strains, including *S. marcescens*, *S. maltophilia*, *B. subtilis*, *K. pneumonia*, and *S. aureus*, with MIC_50_ values of 362 ± 0.13, 413 ± 0.49, 390 ± 0.37, 263 ± 0.82, and 483 ± 0.43 μg/ml, respectively. The ROE, ROH, and ROB extracts also showed good to moderate inhibition against most of the bacterial strains as presented in Table 4. These activities could be attributed to the combined effects of diverse classes of secondary metabolites.

**TABLE 4:** Antibacterial activities of *R. odorata* extract/fractions.

	% Inhibition [MIC_50_ (µg/mL ± S.D.^a^)]									
Sample codes	*E. coli*	*S. aureus*	*C. Koseri*	*K. Pneumonia*	*P. aeruginosa*	*P. mirabilis*	*M. morganii*	*B. subtilis*	*S. maltophilia*	*S. marcescens*
ROE	39 ± 0.4^b^	70 ± 0.83^b^ (286 ± 0.65)	26 ± 0.62^e^	42 ± 0.44^d^	34 ± 0.58^e^	7 ± 0.32^d^	67 ± 0.58^b^ (512 ± 0.22)	88 ± 0.40^b^ (137 ± 0.66)	34 ± 0.57^e^	96 ± 0.31^a^ (55 ± 0.46)
ROH	36 ± 0.61^c^	34 ± 0.61^d^	29 ± 0.86^d^	26 ± 0.45^e^	38 ± 0.35^d^	9 ± 0.60^e^	50 ± 0.57^c^ (503 ± 0.47	85 ± 0.71^c^ (173 ± 0.36)	50 ± 0.41^b^ (482 ± 0.39)	59 ± 0.83^c^ (381 ± 0.72)
ROEA	4 ± 0.44	51 ± 0.29^c^ (483 ± 0.43)	34 ± 0.21^b^	77 ± 0.24^b^ (263 ± 0.82)	39 ± 0.86^b^	43 ± 0.76^c^	44 ± 0.64^d^	57 ± 0.52^d^ (390 ± 0.37)	53 ± 0.95^c^ (413 ± 0.49)	63 ± 0.95^d^ (362 ± 0.13)
ROB	14 ± 0.45^e^	31 ± 0.65^e^	36 ± 0.28^c^	52 ± 0.43^c^ (467 ± 0.24)	8 ± 0.70^c^	26 ± 0.84^b^	51 ± 0.60^d^ (481 ± 0.71)	50 ± 0.64^e^ (495 ± 0.12)	46 ± 0.48^d^	54 ± 0.64^e^ (412 ± 0.59)
Ceftriaxone (references)	84 ± 0.29^a^ (325 ± 0.62)	88 ± 0.44^a^ (130 ± 0.22)	84 ± 0.37^a^ (275 ± 0.61)	85 ± 0.35^a^ (242 ± 0.71)	88 ± 0.32^a^ (121 ± 0.31)	84 ± 0.46^a^ (209 ± 0.43)	87 ± 0.58^a^ (135 ± 0.44)	89 ± 0.60^a^ (121 ± 0.54)	86 ± 0.37^a^ (152 ± 0.83)	89 ± 0.41^b^ (75 ± 0.51)

^a^Results are represented as mean ± S.D., with numerous superscripts a–e that are considerably different from one another (*p* ≤ 0.05).

### 3.4 Antioxidant activity

**FIGURE 1:**
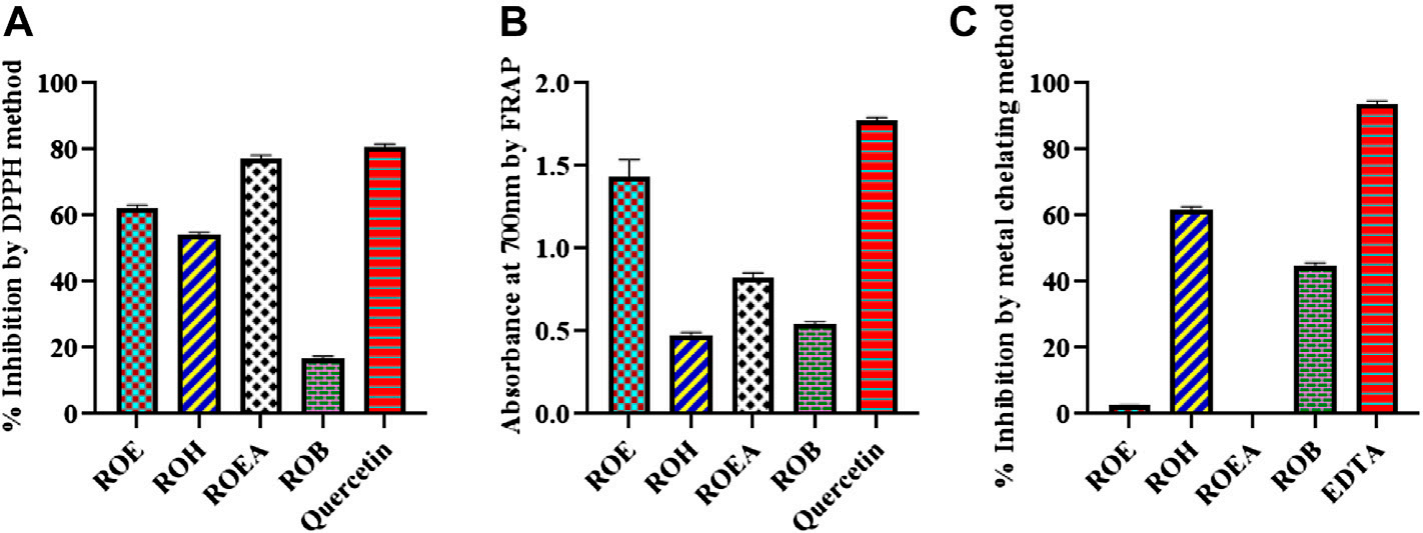
Graphical representation of the antioxidant activity through DPPH **(A)**, FRAP **(B)**, and metal chelating assay **(C)** of aerial extract/fractions of *R. odorata*.

The antioxidant potential of the *R. odorata* was evaluated through DPPH radical scavenging activity, FRAP, and metal-chelating capacity methods. The ability of antioxidants to donate hydrogen is assumed to be the reason for their action on DPPH (Baumann, 1979). Our results with the DPPH assay revealed that ROEA showed the highest antioxidant potential (76.31% ± 1.86%) followed by the ROE (62.13% ± 0.62%) and ROH (54.10% ± 0.70%), while ROB showed the weak activity (16.48% ± 0.87%) (Figure 1; Table 5). The higher radical scavenging activity of ROEA may be due to higher amounts of phenolic compounds in this extract as phenolic compounds possess hydrogen-donating abilities (Khouya et al., 2015; Labiad et al., 2017). Second, good radical scavenging activity of the extracts can further be justified with the presence of high flavonoid contents (Baumann, 1979; Huang et al., 2005), which are also a subclass of phenolics. Characterized by their absorbance values, the ROE extracts showed the highest FRAP activity [1.32 ± 0.01 (absorbance at 700 nm ± S.D.)] as compared to all other extracts, which was comparable with the standard compound quercetin (1.76 ± 0.02), whereas all other extracts exerted lesser inhibition (Table 5). The presence of higher phenolic contents in ROE extract contributes to its good inhibitory activity and several investigations have shown that phenolic-rich extracts are better antioxidants when evaluated by FRAP activity (Wojdyło et al., 2007; Firuzi et al., 2010; Mashkor, 2014; Sethi et al., 2020). Some of the plant metabolites have been reported to possess the ability to bind with metal ions, which forms chelation with harmful metal ions and makes complex structures that can be easily eliminated from the body. Therefore, the metal-chelating capacity of the plant extracts herein was measured and ROH showed a greater metal-chelating capacity with a value of 62.61% ± 1.01% followed by ROB (44.35% ± 1.83%), whereas ROE and ROEA were inactive.

**TABLE 5:** Antioxidant activities of the *R. odorata* extract/fractions.

Sample codes	DPPH radical scavenging activity (inhibition % ± S.D.^a^)	FRAP (absorbance at 700 nm ± S.D.)^b^	Metal-chelating capacity (inhibition % ± S.D.)
ROE	62.13 ± 0.62	1.32 ± 0.01	2.53 ± 0.38
ROH	54.10 ± 0.70	0.48 ± 0.01	62.61 ± 1.01
ROEA	76.31 ± 1.86	0.80 ± 0.02	NA^c^
ROB	16.48 ± 0.87	0.53 ± 0.02	44.35 ± 1.83
Quercetin^d^	80.89 ± 0.54	1.76 ± 0.02	
EDTA^e^			94.57 ± 0.62

^a^ Standard deviation (*n*: 3).

^b^ Higher absorbance indicates higher antioxidant activity in FRAP.

^c^ No activity.

^d^ Reference for DPPH, radical scavenging activity at 1000 μg/ml.

^e^ Reference for metal-chelating capacity at 2000 μg/ml. Extract/fractions concentration for DPPH, FRAP, and metal chelating assay remained at 5 mg/ml in methanol.

### 3.5 Enzyme inhibition studies

**FIGURE 2:**
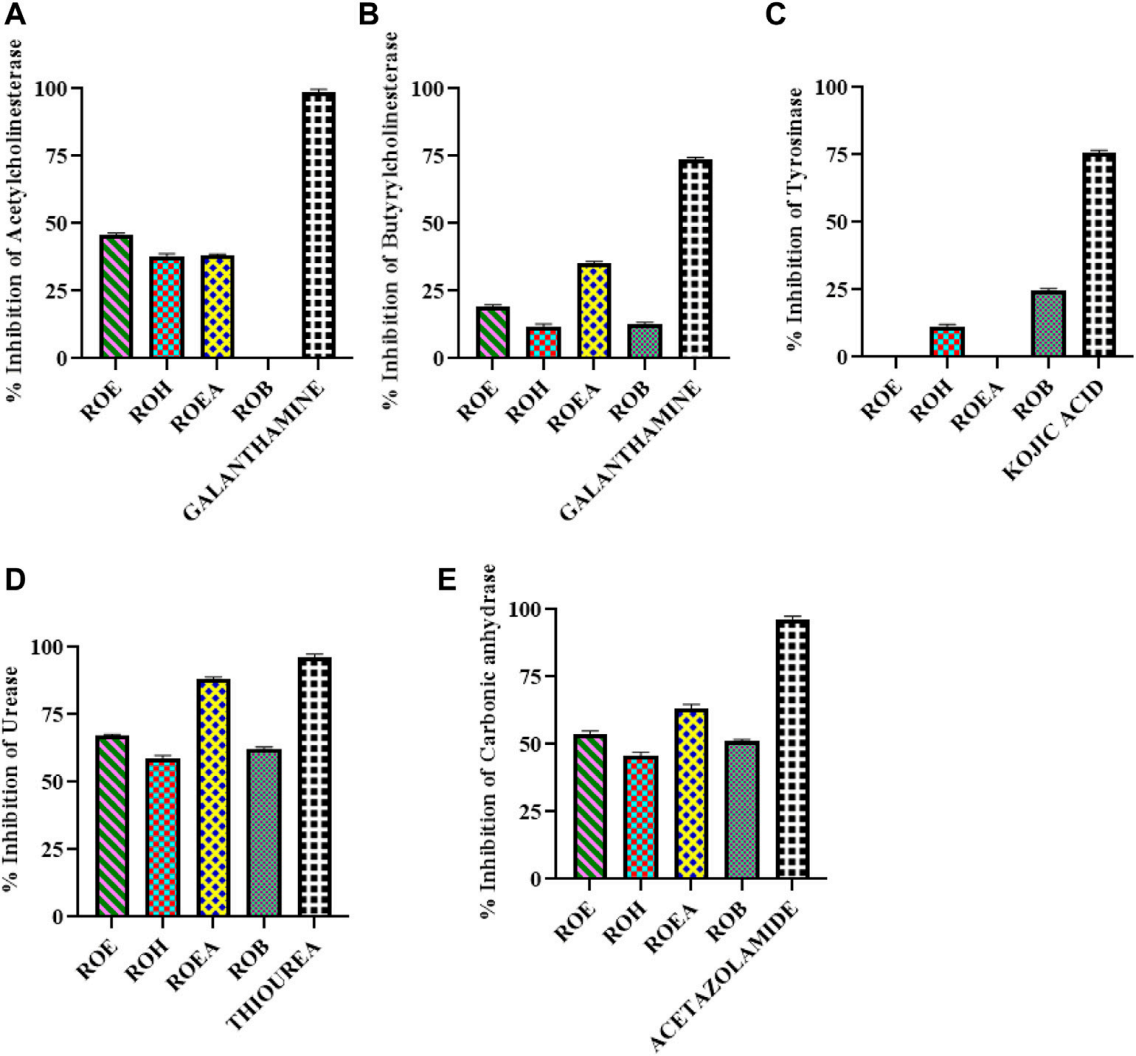
Graphical representation of AChE **(A)**, BChE **(B)**, TYR **(C)**, urease **(D)**, and CA **(E)** inhibition of aerial extract/fractions of *R. odorata*.

Enzyme inhibitory potential of *R. odorata* (Figure 2; Table 6) was also assessed against five enzymes of clinical significance, which include AChE, BChE, TYR, urease, and CA. All of the test samples were found active against urease and CA, while ROEA extract had a maximum inhibition (IC_50_ values of 2.78 ± 0.42 mg/ml and 3.95 ± 0.13 mg/ml, respectively) against both the enzymes. The ROE, ROB, and ROH fractions were next in line of activity level (Table 6). In this assay, the standard compounds thiourea and acetazolamide showed IC_50_ values of 0.54 ± 0.11 mg/ml and 0.54 ± 0.08 mg/ml, respectively.

**TABLE 6:** Enzyme inhibitory activities of *R. odorata* extract/fractions.

Sample codes	AChE (inhibition % ± S.D.^a^) At 200 µg/mL	BChE (inhibition % ± S.D.) at 200 μg/ml	TYR (inhibition % ± S.D.) at 100 μg/ml	Urease (inhibition % ± S.D.) at 5,000 μg/ml, IC_50_ (mg/ml)	CA (inhibition % ± S.D.) at 5,000 μg/ml, IC_50_ (mg/ml)
ROE	45.85 ± 2.27	19.72 ± 2.52	NA^b^	67 ± 0.43 (IC_50_ : 3.94 ± 0.20)	52 ± 2.13 (IC_50_ : 4.69 ± 0.35)
ROH	38.92 ± 4.34	10.5 ± 0.15	10.52 ± 0.94	59 ± 0.62 (IC_50_ : 4.06 ± 0.18)	44 ± 1.57
ROEA	37.67 ± 1.22	34.69 ± 3.56	NA	88 ± 0.39 (IC_50_ : 2.78 ± 0.42)	64 ± 0.83 (IC_50_ : 3.95 ± 0.13)
ROB	NA	11.48 ± 3.01	24.52 ± 2.46	61 ± 0.74 (IC_50_ : 4.08 ± 0.21)	51 ± 0.82 (IC_50_ : 4.82 ± 0.07)
Galantamine^c^	97.11 ± 1.26 (IC_50_: 0.68 ± 0.05 μg/ml)	72.88 ± 2.61 (IC_50_: 42.85 ± 5.72 μg/ml)			
α-Kojic acid^d^			76.58 ± 0.85 (IC_50_: 52.42 ± 2.67 μg/ml)		
Thiourea^e^				97 ± 0.39 (IC_50_ : 0.54 ± 0.11)	
Acetazolamide^f^					96 ± 0.51 (IC_50_ : 0.54 ± 0.08)

^a^ Standard deviation (*n*: 3).

^b^ No activity.

^c^ Reference (100 μg/ml) for AChE and BChE inhibition.

^d^ Reference (200 μg/ml) for TYR inhibition.

^e^ Reference (0.375 mM) for urease inhibition.

^f^ Reference (0.1 mM) for CA inhibition.

### 3.6 Antiviral activity

**FIGURE 3:**
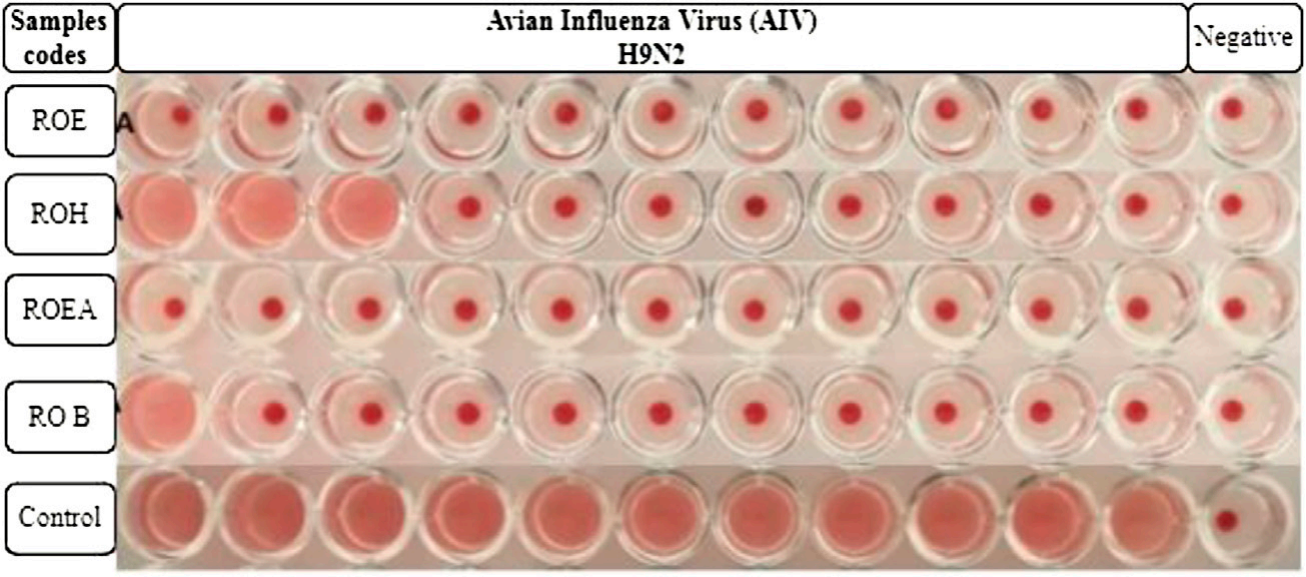
Antiviral activity of all of the extracts against avian influenza virus (AIV) H9N2.

**FIGURE 4:**
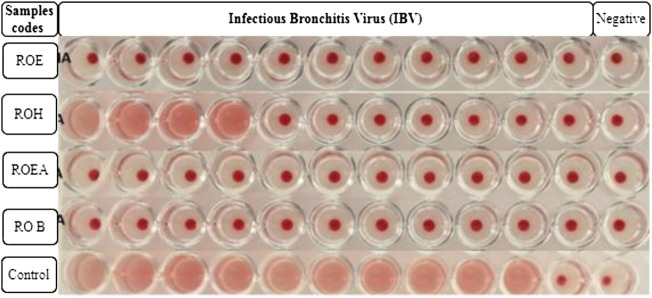
Antiviral activity of all of the extracts against infectious bronchitis virus (IBV).

**FIGURE 5:**
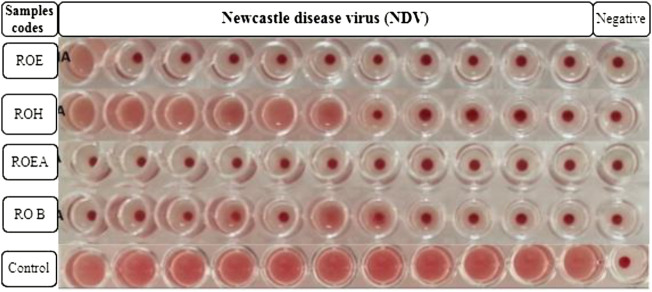
Antiviral activity of all of the extracts against Newcastle disease virus (NDV).

**FIGURE 6:**
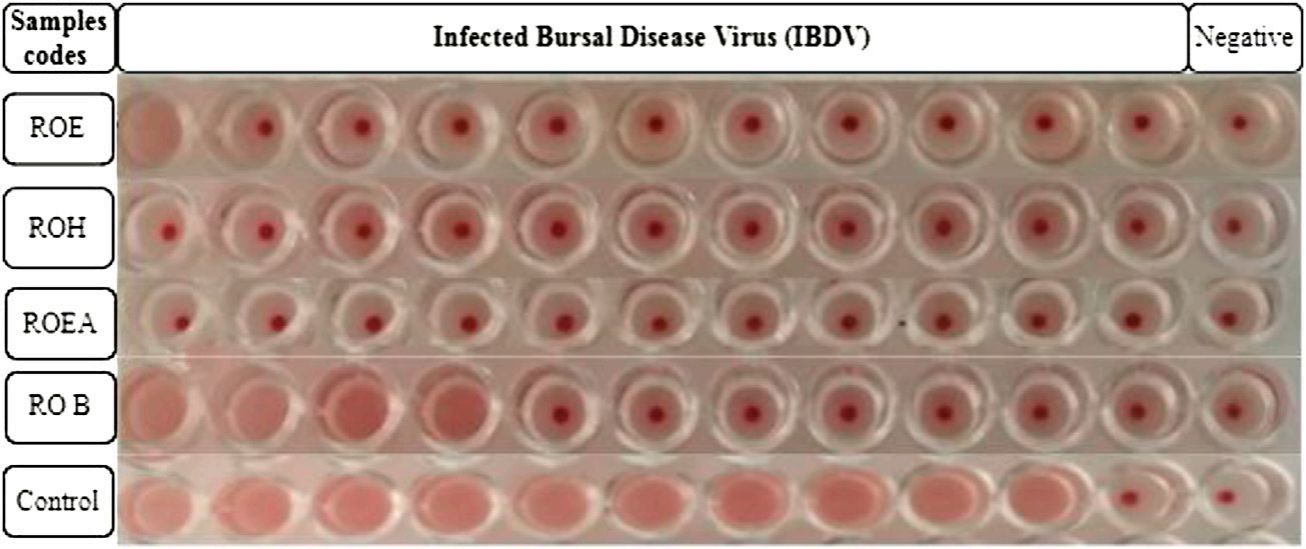
Antiviral activity of all of the extracts against infected bursal disease virus (IBDV).

COVID-19, a recent global pandemic produced by SARS-CoV-2, has wreaked havoc on communities all over the world. There is no specific drug to treat COVID-19 at the moment for the pandemic; therefore, it is very crucial to look into all possibilities for developing a much-needed therapeutic medication against SARS-CoV-2 (Stasi et al., 2020). As a result, antiviral research on infectious bronchitis virus (IBV) is very important, which has properties comparable to the coronavirus (Wang et al., 2020) and should be useful as a target microorganism in the development of novel antiviral medicines. Furthermore, infected bursal disease virus (IBDV) is also a devastating virus due to the lack of antiviral brands on the market. Several studies have discovered evidence of the usage of therapeutic herbs to treat the deadly IBDV (Pant et al., 2012). IBDV is often known as HIV for poultry since it causes immunosuppression (HIV causes AIDS in humans) (Fauci, 2003). Consequently, an antiviral study of all of the fractions of *R. odorata* extract was carried out against four viruses, including IBV and IBDV, and significant results were obtained as shown in Table 7. The experiment was performed with a concentration of 25 mg/ml in methanol for each extract/fraction; PBS was used as a –ve control, while acyclovir was used as a +ve control. The numbers in Table 7 indicate the titer score. The extracts’ efficacy was measured in terms of viral growth; therefore, the titer score is directly proportional to the number of viral particles (Musaddiq et al., 2020). In this work, the extract and fractions showed good antiviral efficacy against all of the target viruses, including avian influenza virus (AIV) H9N2, IBV, Newcastle disease virus (NDV), and (IBDV), with very little viral titer growth (Table 7; Figures 3–6). ROH and ROB extracts showed a weaker antiviral activity with a viral titer of 64 (NDV) and 16 (IBDV), whereas ROE expressed good activity with a viral titer of 0–2 against all of the viruses and ROEA displayed the highest activity with no viral titer. These activities were also correlated with the phenolic and flavonoid contents. Therefore, it is concluded that *R. odorata* may have a notable potential as an antiviral agent or may pave the way for the development of novel antiviral compounds derived from this plant to battle viral infections.

**TABLE 7:** Antiviral activities of *R. odorata* extract/fractions against H9, IBV, NDV, and IBDV viral strains.

Sample codes	H9N2	Control	IBV	Control	NDV	Control	IBDV	Control
ROE	00	2048	00	1024	02	2048	02	1024
ROH	08	2048	16	1024	64	2048	00	1024
ROEA	00	2048	00	1024	00	2048	00	1024
ROB	02	2048	00	1024	00	2048	16	1024

### 3.7 Hemolytic activity

In order to check the cytotoxic activity of the plant extract and its fractions, the hemolysis assay, which causes lysis and cell death by damaging the cytoplasmic membrane of red blood cells, was performed. All of the extracts showed very weak hemolytic activity as presented in Table 8. This study indicated that the plant is safe to be used as a potential medicinal herb.

**TABLE 8:** Cytotoxic and thrombolytic activities of *R. odorata* extract/fractions.

Sample codes	Hemolysis %	Thrombolytic activity %
ROE	10.1	3.8
ROH	7.2	3.8
ROEA	8.7	3.8
ROB	2.2	13.5
Triton × 100 (control)	93.5	—
PBS	0.0	—
Streptokinase (references)		91.1

### 3.8 Thrombolytic activity

A major problem among non-communicable diseases is failure of hemostasis, which causes thrombus (blood clot) development and may cause a partial or complete blockage in small vessels of the blood circulatory system. This arterial blockage can result in life-threatening thrombotic disorders, including acute myocardial or cerebral infarction leading to death (Merlyn Keziah and Subathra Devi, 2018). Thrombolytic medicines such as streptokinase, alteplase, anistreplase, urokinase, and tissue plasminogen activator (tPA) are commonly used to dissolve thrombus. Most of them are synthetic and have side effects. Additionally, people from developing countries have very little access to modern health facilities and they keenly depend on the local medicinal system (Li et al., 2020). Therefore, there is a critical need to explore indigenous sources for novel, safer, and more effective thrombolytic agents. In the current work, all extracts were checked for their thrombolytic activity and ROB was found to be the most active fraction among all extracts (Table 8). ROB could have glycosides of various metabolites, which predicts that highly polar metabolites could be responsible for this activity.

### 3.9 Docking studies

Molecular docking provides a scaffold to understand the biomolecular interactions between potential drugs and receptor proteins. The docking of compounds with potential drug targets helps in understanding the mechanistic approach of how it binds with its receptor proteins through noncovalent interactions and gives an idea about the stability of ligand–receptor complex along with potential efficacy and specificity (Rohs et al., 2005; Guedes et al., 2014). Therefore, docking studies of some compounds were performed, which were selected on the base concentration with respect to class, and results are presented in Table 9 and Table 10.

**TABLE 9:** Binding free energy and inhibition constants of docked complexes against CA and urease enzymes.

Ligands	CA		Urease	
	Binding free energy	Estimated inhibition constant	Binding free energy	Estimated inhibition constant
^a^Control	−6.65	13.37 uM	−3.11	5.27 mM
Caryoptosidic acid	−5.73	63.50 uM	−5.07	191.93 uM
Methymycin	−7.90	1.61 uM	−2.28	21.34 mM
Minabeolide-1	−9.59	93.81 nM	−6.85	9.52 uM
Minabeolide-8	−9.57	96.19 nM	−9.00	253.77 nM
Norselic acid	−7.80	1.92 uM	−5.37	116.12 uM
Piperolactam A	−7.54	2.95 uM	−6.30	24.09 uM

^a^ For CA, acetazolamide is used as a reference, while for urease, thiourea is used as a reference compound for docking studies.

**TABLE 10:** Binding interaction patterns of best-docked complexes for urease.


Ligands	Bond category	Bond distance	Bond type	Interactions			
				Residue name and groups	From chemistry	Residue name and groups	To chemistry
Interaction patterns for urease enzyme							
Acetazolamide	Hydrogen bond	2.84853	Conventional hydrogen bond	A:SER65:HG:B	C-H	A:AZM263:O2	H-Acceptor
		2.77235		A:GLN92:HE22		A:AZM263:N1	
		2.76614		A:THR199:HG1		A:AZM263:N2	
	Electrostatic	3.3013	Pi–Cation	A:ZN262:ZN	Positive	A:AZM263	
	Hydrogen bond	3.60807	Pi–Donor hydrogen bond	A:AZM263:N1	H-Donor	A:HIS94	Pi-Orbitals
	Other	3.76389	Pi–Sulfur	A:AZM263:S1	Sulfur	A:HIS94	Pi-Orbitals
	Hydrophobic	4.22618	Pi–Pi Stacked	A:HIS94	Pi-Orbitals	A:AZM263	Pi-Orbitals
	Hydrophobic	4.1873	Alkyl	A:AZM263:C4	Alkyl	A:VAL143	Alkyl
		4.35352		A:AZM263:C4		A:LEU198	
		4.01591		A:AZM263:C4		A:VAL207	
	Hydrophobic	4.71578	Pi–Alkyl	A:TRP209	Pi-Orbitals	A:AZM263:C4	Alkyl
		4.13746		A:TRP209		A:AZM263:C4	
Minabeolide-1	Hydrophobic	3.60303	Pi–Sigma	LIG:C	C-H	A:HIS94	Pi-Orbitals
	Hydrophobic	4.86143	Alkyl	A:LEU198	Alkyl	LIG	Alkyl
		4.62259		LIG:C		A:VAL135	
		4.95599		LIG:C		A:PRO202	
		3.6865		LIG:C		A:VAL143	
		4.56869		LIG:C		A:LEU198	
		3.55419		LIG:C		A:VAL207	
		3.26595		LIG:C		A:VAL121	
		4.78682		LIG:0043		A:LEU141	
		4.0555		LIG:C		A:VAL143	
		4.74499		LIG:C		A:LEU198	
	Hydrophobic	5.00704	Pi–Alkyl	A:HIS94	Pi-Orbitals	LIG	Alkyl
		4.96148		A:HIS96		LIG:C	
		5.17453		A:PHE131		LIG:C	
		5.3508		A:PHE131		LIG	
		4.53749		A:TRP209		LIG:C	
		4.02569		A:TRP209		LIG:C	
Minabeolide-8	Hydrogen bond	3.16814	Carbon hydrogen bond	A:HIS64:CE1:B	H-Donor	LIG:O	H-Acceptor
	Hydrophobic	3.7703	Pi–Sigma	LIG:C	C-H	A:HIS94	Pi-Orbitals
	Hydrophobic	3.50985	Alkyl	LIG:C	Alkyl	A:VAL143	Alkyl
		5.12415		LIG:C		A:LEU198	
		4.33114		LIG:C		A:PRO202	
	Hydrophobic	4.29031	Pi–Alkyl	A:HIS94	Pi-Orbitals	LIG	Alkyl
		5.17523		A:HIS94		LIG	
		5.00028		A:HIS96		LIG	
		4.66563		A:HIS96		LIG:C	
		5.1792		A:HIS119		LIG	
		5.18126		A:TRP209		LIG:C	
		4.08923		A:TRP209		LIG:C	
Interaction patterns for urease enzyme							
Thiourea	Hydrogen bond	2.23669	Conventional hydrogen bond	A:THR520:HN	H-Donor	A:TOU101:S	H-Acceptor
		2.9712		A:HIS545:HD1		A:TOU101:S	
		2.82557		A:TOU101:N2		A:ILE518:O	
	Other	4.18268	Pi–Sulfur	A:TOU101:S	Sulfur	A:HIS545	Pi-Orbitals
Minabeolide-1	Hydrogen bond	2.74059	Conventional hydrogen bond	A:HIS519:HE2	H-Donor	LIG:O	H-Acceptor
	Other	2.71871	Sulfur–X	A:MET588:SD	Sulfur	LIG:O	O,N,S
	Hydrophobic	3.05226	Alkyl	A:ALA440	Alkyl	LIG:C	Alkyl
		4.42443		A:ALA440		LIG	
		4.94936		A:ALA636		LIG	
		4.4799		LIG:C		A:ARG439	
		3.77426		LIG:C		A:MET588	
		4.70633		LIG:C		A:MET637	
	Hydrophobic	4.26942	Pi–Alkyl	A:HIS492	Pi-Orbitals	LIG:C	Alkyl
		4.1535		A:HIS593		LIG	
Minabeolide-8	Hydrophobic	4.47773	Alkyl	A:ALA440	Alkyl	LIG	Pi-Orbitals
		4.46281		LIG:C		A:MET588	
		5.31266		LIG		A:MET588	
		4.54221		LIG:C		A:ARG439	
	Hydrophobic	4.04795	Pi–Alkyl	A:HIS593	Pi-Orbitals	LIG	Alkyl

#### 3.9.1 Postdock analysis

**FIGURE 7:**
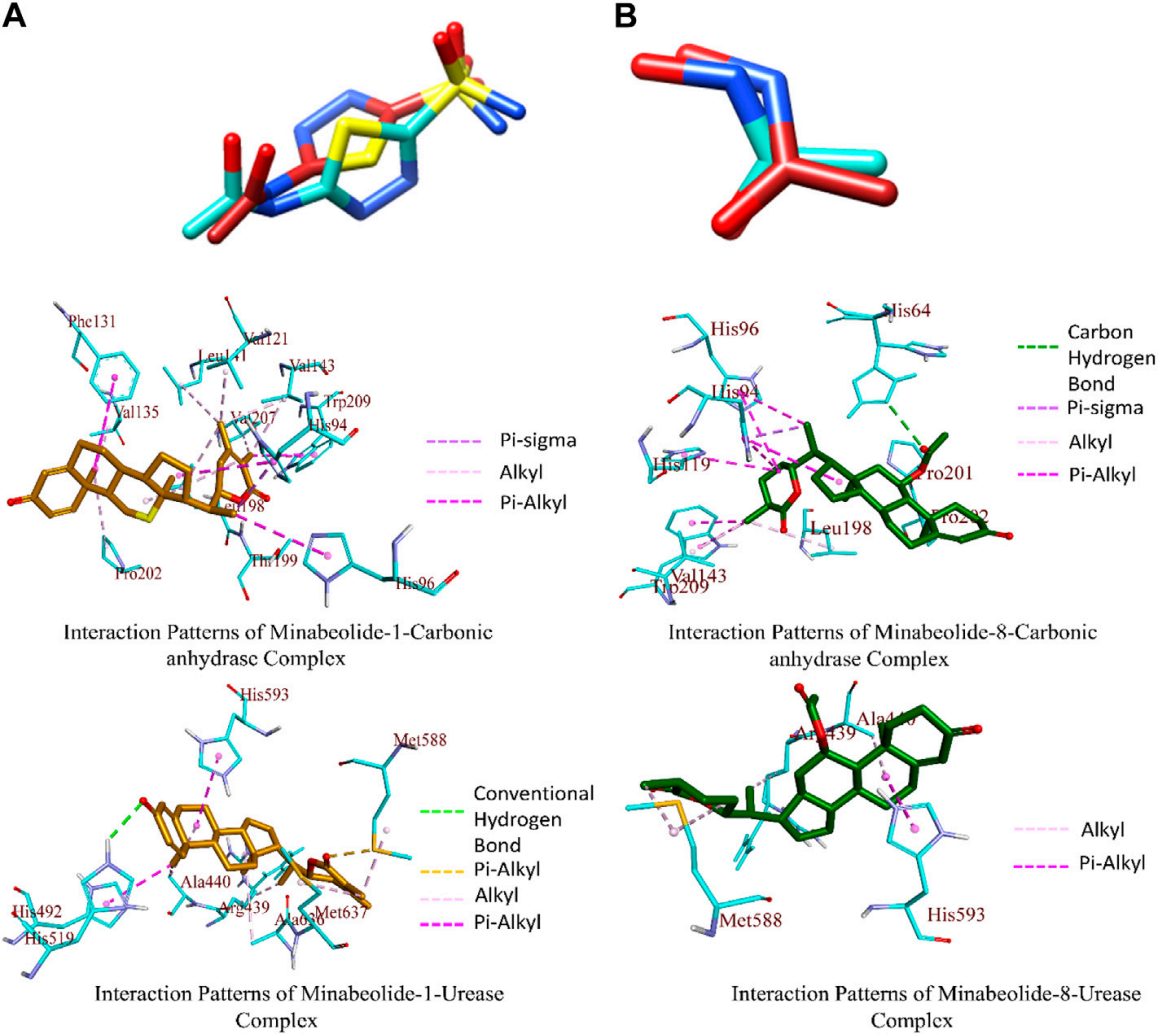
**(A)** Superimposed re-docked acetazolamide (brick red) on co-crystallized acetazolamide (turquoise colored). **(B)** Superimposed re-docked thiourea (brick red) on co-crystallized thiourea (turquoise colored). Binding interaction patterns for carbonic anhydrase and urease in complex with ligands having high affinity toward respective enzymes.

**FIGURE 8:**
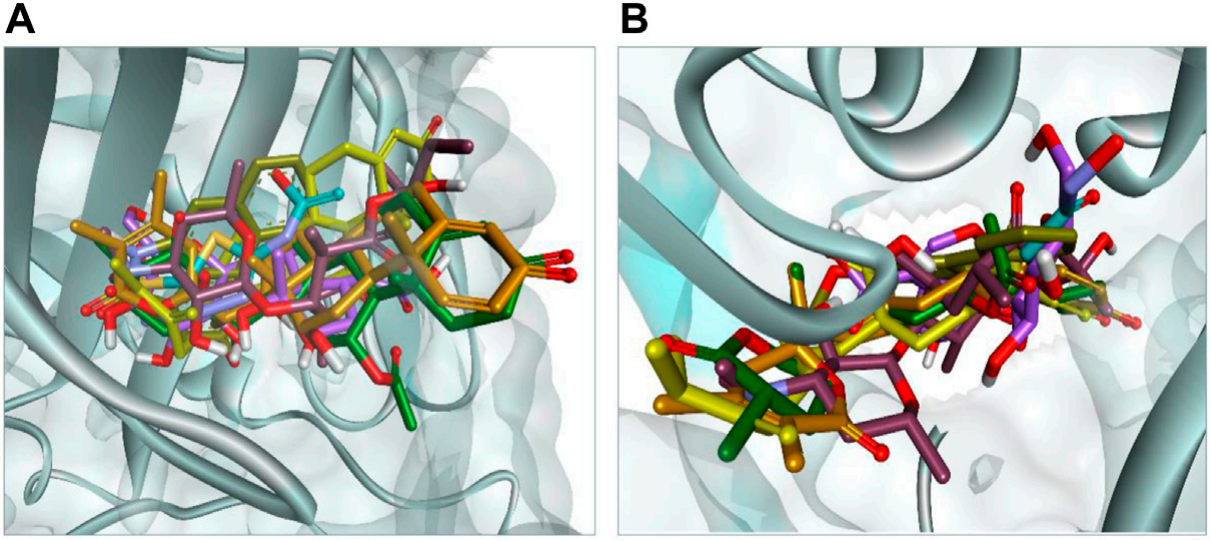
**(A)** Binding pose of top-ranked docked conformation of all ligands compared on co-crystallized acetazolamide (turquoise colored) with in active site of carbonic anhydrase. **(B)** Binding pose of top-ranked docked conformation of all ligands compared on co-crystallized thiourea (turquoise colored) with in active site of urease.

The docking results were analyzed on the basis of RMSD clustering, and top-ranked clusters were identified. Thiourea was used as the reference for urease docking while acetazolamide was the reference for CA. The validation of the docking protocol was performed through the redocking of co-crystallized ligands for their respective enzymes. The redocking of co-crystallized ligands showed that acetazolamide showed an RMSD of 1.203 A° from co-crystallized ligand (Figure 7A) while thiourea exhibited an RMSD of 0.621 A° (Figure 7B). The re-docking results validate that the docking protocol as RMSD within the range of 2 A° is acceptable (Kramer et al., 1999). The comparison of the binding potential of compounds to the reference compound revealed that all of the compounds except methymycin showed an increased binding affinity against urease than thiourea, while the binding potential of caryoptosidic acid for CA is less than that of the reference. The docking studies revealed that these compounds have a better binding potential for urease and CA (Table 9). Docking results revealed that compounds minabeolide-1 and -8 exhibited binding free energy of −9.59 and −9.57 kcal/mol, respectively, against CA, while acetazolamide as the reference has a binding potential of −6.65 kcal/mol. This indicates that these two compounds interact more efficiently with active site residue of carbonic anhydrase than acetazolamide. The clustering of ligands showed that all ligands cluster within the active site of CA (Figure 8). These two compounds outperformed in docking studies against urease and showed the binding potential of −6.85 and −9.00 kcal/mol, respectively. All ligands were visualized on the co-crystallized ligand of carbonic anhydrase (Figure 8A) and urease (Figure 8B) *via* Discovery Studio Visualizer in order to obtain an insight if these ligands bind at the same site. For carbonic anhydrase, all ligands superimposed on acetazolamide present within the active site of the crystal structure and interacted with amino acid residues within active site of the enzyme, as shown in Figure 8A. The same pattern was observed for urease enzyme (Figure 8B): the top docked conformation of all ligands was bound at the same active site and found to be superimposed on thiourea when visualized. The top binding poses of all ligands were clustered within the active site of the receptor protein (Figure 7).

The top-ranked docked complexes were further analyzed through the Discovery Studio Visualizer to identify the binding interaction patterns. The minabeolide-1 mediated three types of hydrophobic interactions with CA, namely, pi–sigma, alkyl, and pi–alkyl. The amino acid residue HIS94 mediated pi–sigma interactions; LEU198, VAL135, PRO202, VAL143, LEU198, VAL207, VAL121, LEU141, and LEU198 mediated alkyl-type interactions, while HIS94, HIS96, PHE131, and TRP209 mediated pi–alkyl interactions involving pi-orbitals of ligand (Figure 7). The analysis of binding interactions for minabeolide-8 exhibited that HIS64 was involved in forming a carbon–hydrogen bond, and pi–sigma bond was mediated by HIS94 (Figure 7). The alkyl and pi–alkyl interactions were also mediated by a number of protein residues provided in Table 10.

The minabeolide-1 and -8 also outperformed in the case of docking studies against urease. The analysis of the binding interaction of these two compounds showed that minabeolide-1 mediated conventional hydrogen bond, hydrophobic interactions, and some other interactions involving sulfur of MET588 residue of proteins, while minabeolide-8 mediated two types of hydrophobic interactions, namely, alkyl and pi–alkyl interactions. The details of interactions, residues involved, and bond distance are provided in Table 10.

## 4 Conclusion

The present study offers a deep insight into the chemical and biological diversity of *R. odorata*. Estimation of total bioactive contents revealed that this plant produces a high amount of phenolics and flavonoids with significant antioxidant activity, which offers the candidature of this plant as a potential ingredient of nutraceuticals and functional foods. The presence of diverse secondary metabolites and a variety of biological activities of the extract and fractions of *R. odorata* substantiates the aforementioned deduction. Since it is the first investigation on *R. odorata*, further studies may unveil this plant as a promising source of antioxidant, antibacterial, antiulcer, diuretic, and antiviral agents. Thus, further *in vivo* studies and toxicity testing on *R. odorata* are strongly recommended to authenticate this plant as a prospective source of natural biologically active agents.

## Data Availability

The original contributions presented in the study are included in the article/Supplementary Materials; further inquiries can be directed to the corresponding authors.
